# 9-Meth­oxy-9-(2-meth­oxy­phen­yl)-9*H*-xanthene

**DOI:** 10.1107/S1600536812037415

**Published:** 2012-09-05

**Authors:** Ayesha Jacobs, Francoise M. Amombo Noa, Jana H. Taljaard

**Affiliations:** a Chemistry Department, Cape Peninsula University of Technology, PO Box 652, Cape Town, 8000, South Africa; b Sasol Technologies, R&D Klasie Havenga Road 1, Sasolburg 1947, South Africa

## Abstract

In the title compound, C_21_H_18_O_3_, the xanthene system and the meth­oxy­phenyl ring are practically orthogonal with a dihedral angle between their mean planes of 89.27 (3)°. The meth­oxy group attached to the phenyl ring makes a C—O—C—C torsion angle of 11.56 (18)°. In the crystal, mol­ecules are linked by C—H⋯O inter­actions into chains along [010]. Weak C—H⋯π inter­actions also occur.

## Related literature

For the synthesis of the parent xanthenol compound 9-(2-meth­oxy­phen­yl)-9*H*-xanthen-9-ol, see: Dilthey *et al.* (1939 ▶). For related inclusion chemistry of 9-(2-meth­oxy­phen­yl)-9*H*-xanthen-9-ol, see: Jacobs *et al.* (2005 ▶, 2007 ▶, 2009 ▶). For related structures, see: Das *et al.* (2007 ▶). For the design of host compounds, see: Weber (1991 ▶) and for a review of C—H⋯O inter­actions, see: Steiner (1997 ▶).
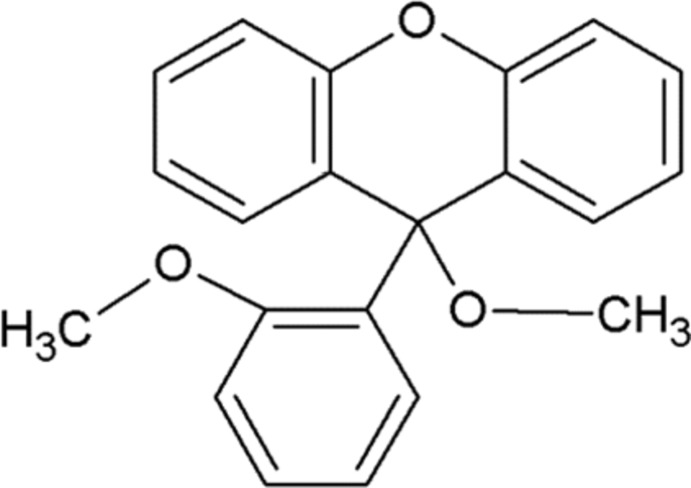


## Experimental

### 

#### Crystal data

C_21_H_18_O_3_
*M* *_r_* = 318.35
Monoclinic, 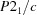

*a* = 8.0665 (6) Å
*b* = 9.7653 (7) Å
*c* = 21.3191 (15) Å
β = 105.560 (2)°
*V* = 1617.8 (2) Å^3^
*Z* = 4
Mo *K*α radiation
μ = 0.09 mm^−1^
*T* = 173 K
0.22 × 0.16 × 0.03 mm

#### Data collection

Bruker Kappa DUO APEXII diffractometer
Absorption correction: multi-scan (*SADABS*; Sheldrick, 1996 ▶) *T*_min_ = 0.833, *T*_max_ = 0.997
15054 measured reflections
4041 independent reflections
2779 reflections with *I* > 2σ(*I*)
*R*_int_ = 0.039

#### Refinement

*R*[*F* ^2^ > 2σ(*F* ^2^)] = 0.043
*wR*(*F* ^2^) = 0.117
*S* = 1.02
4041 reflections
219 parameters
H-atom parameters not refined
Δρ_max_ = 0.27 e Å^−3^
Δρ_min_ = −0.19 e Å^−3^

### 

Data collection: *APEX2* (Bruker, 2005 ▶); cell refinement: *SAINT-Plus* (Bruker, 2005 ▶); data reduction: *SAINT-Plus* and *XPREP* (Bruker, 2005 ▶); program(s) used to solve structure: *SHELXS97* (Sheldrick, 2008 ▶); program(s) used to refine structure: *SHELXL97* (Sheldrick, 2008 ▶); molecular graphics: *X-SEED* (Barbour, 2001 ▶); software used to prepare material for publication: *SHELXL97* and *PLATON* (Spek, 2009 ▶).

## Supplementary Material

Crystal structure: contains datablock(s) I, global. DOI: 10.1107/S1600536812037415/hg5247sup1.cif

Structure factors: contains datablock(s) I. DOI: 10.1107/S1600536812037415/hg5247Isup2.hkl

Supplementary material file. DOI: 10.1107/S1600536812037415/hg5247Isup3.cml

Additional supplementary materials:  crystallographic information; 3D view; checkCIF report

## Figures and Tables

**Table 1:** Hydrogen-bond geometry (Å, °) *Cg* is the centroid of the C14–C19 ring.

*D*—H⋯*A*	*D*—H	H⋯*A*	*D*⋯*A*	*D*—H⋯*A*
C17—H17⋯O1^i^	0.95	2.55	3.303 (2)	136
C20—H20*C*⋯*Cg* ^ii^	0.98	2.82	3.6802 (16)	147

Symmetry codes: (i) 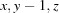; (ii) 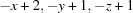.

**Fig. 1.**
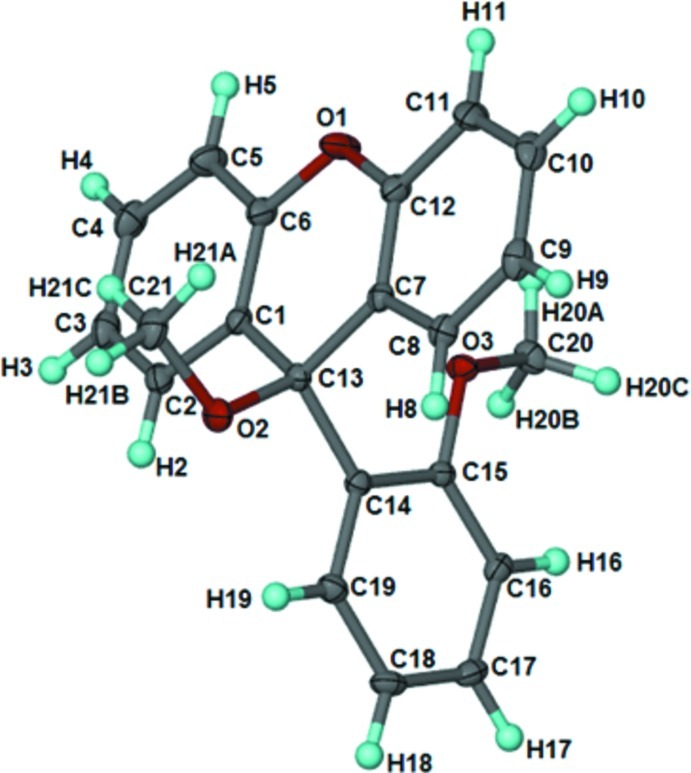
Thermal ellipsoid plot of 9-methoxy-9-(2-methoxyphenyl)-9H-xanthene at the 30% probability level indicating the atomic numbering scheme.

**Fig. 2.**
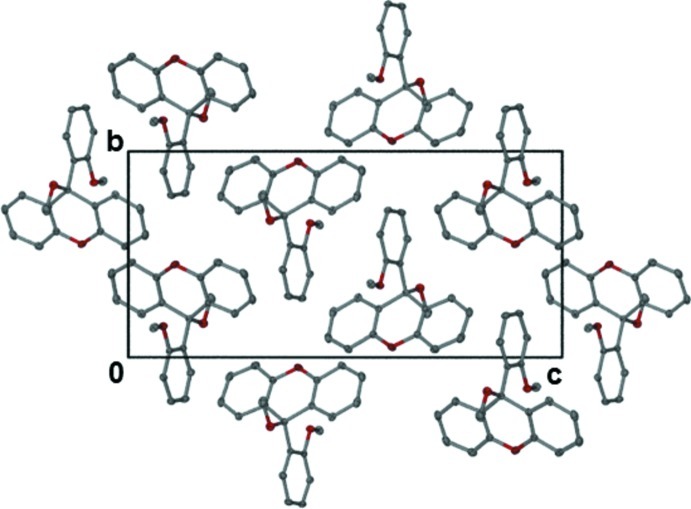
Packing diagram of 9-methoxy-9-(2-methoxyphenyl)-9H-xanthene down [100].

**Fig. 3.**
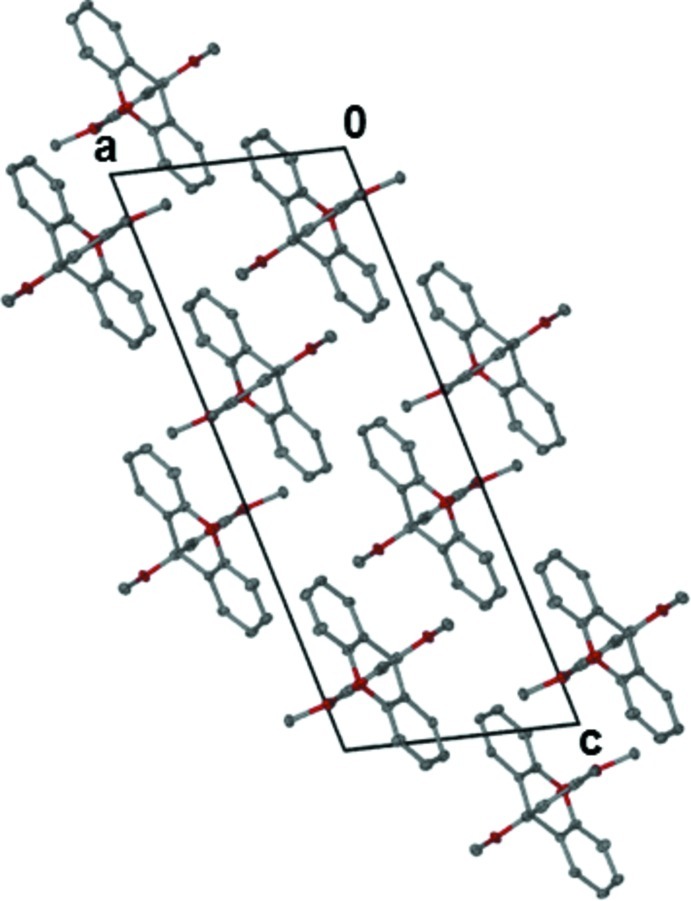
Packing diagram of 9-methoxy-9-(2-methoxyphenyl)-9H-xanthene down [010].

## supplementary crystallographic information

### Comment

The starting material, 9-(2-methoxyphenyl)-9*H*-xanthen-9-ol, was synthesized by reported methods (Dilthey *et al.*, 1939). Xanthenol compounds have been used extensively in host–guest chemistry as versatile hosts for the inclusion of small organic guests (Jacobs *et al.*, 2005), solvent free reactions (Jacobs *et al.*, 2009) and guest exchange experiments (Jacobs *et al.*, 2007). This class of compounds conforms to Weber's rules (Weber, 1991) for efficient hosts in that they are bulky and contain functionalities that can participate in hydrogen bonding. Charge delocalization into the adjacent aromatic rings of the xanthene moiety can stabilize a cationic centre at C13 (Fig. 1), facilitating nucleophilic attack. The loss of the hydroxyl group yields a compound without a strong hydrogen bond donor.

The structure crystallized in *P*2_1_/*c* with one molecule in the asymmetric unit. Short C—H··· O contacts [C···O = 3.303 (2) Å and C—H···O = 136°] link adjacent molecules into anti-parallel chains along [010] (Fig. 2). Similar resonance assisted weak hydrogen bonding has been described (Steiner, 1997) for polarisable π-bond systems. An intramolecular C—H··· O contact [C···O = 2.663 (1) Å and C—H··· O = 102°] gives rise to a torsion angle O2—C13—C14—C19 = -0.14 (17)°.

Weaker C—H···π interactions include C20···π(C14—C19) = 3.680 Å and an intramolecular C21···π(O1—C13) = 2.974 Å. The shortest π–π contact of 4.034 Å is an intramolecular edge to face interaction between π(O1—C13) and π(C14—C19). Ten xanthene derivatives were synthesized from the parent compound 9-phenyl-9*H*-xanthene-9-ol and selected ketones (Das *et al.*, 2007). C—H···π, C—H···O and π–π interactions dominated the structures with typical distances of 2.664 Å, 3.378 Å and 4.691 Å respectively.

The packing diagram down [010] is shown in Fig. 3. The xanthene ring and the methoxyphenyl moiety are practically orthogonal with a dihedral angle between the least squares planes of 89.27 (3)°. The methoxy moiety attached to the phenyl ring deviates from the C14—C19 plane with a resultant C20—O3—C15—C16 torsion angle of 11.56 (18)°.

### Experimental

A crystal of 9-methoxy-9-(2-methoxyphenyl)-9*H*-xanthene was prepared serendipitously by slow evaporation of a dilute solution of 9-(2-methoxyphenyl)-9*H*-xanthen-9-ol and theophylline in a 50:50 mixture of methanol/chloroform.

### Refinement

The aromatic and methyl hydrogen atoms were geometrically constrained, with C—H distances fixed at 0.95 Å and 0.98 Å respectively. For the aromatic H atoms *U*_iso_(H) = 1.2*U*_eq_(C_aryl_) and for the methyl H atoms *U*_iso_(H) = 1.5*U*_eq_(C_methyl_).

### Crystal data

**Table:**

C_21_H_18_O_3_	*F*(000) = 672
*M**_r_* = 318.35	*D*_x_ = 1.307 Mg m^−^^3^
Monoclinic, *P*2_1_/*c*	Mo *K*α radiation, λ = 0.71073 Å
Hall symbol: -P 2ybc	Cell parameters from 15054 reflections
*a* = 8.0665 (6) Å	θ = 0.1–28.4°
*b* = 9.7653 (7) Å	µ = 0.09 mm^−^^1^
*c* = 21.3191 (15) Å	*T* = 173 K
β = 105.560 (2)°	Block, colourless
*V* = 1617.8 (2) Å^3^	0.22 × 0.16 × 0.03 mm
*Z* = 4	

### Data collection

**Table:**

Bruker Kappa DUO APEXII diffractometer	4041 independent reflections
Radiation source: fine-focus sealed tube	2779 reflections with *I* > 2σ(*I*)
Graphite monochromator	*R*_int_ = 0.039
1.2° φ scans and ω scans	θ_max_ = 28.4°, θ_min_ = 2.0°
Absorption correction: multi-scan (*SADABS*; Sheldrick, 1996)	*h* = −10→10
*T*_min_ = 0.833, *T*_max_ = 0.997	*k* = −13→13
15054 measured reflections	*l* = −28→28

### Refinement

**Table:**

Refinement on *F*^2^	Primary atom site location: structure-invariant direct methods
Least-squares matrix: full	Secondary atom site location: difference Fourier map
*R*[*F*^2^ > 2σ(*F*^2^)] = 0.043	Hydrogen site location: inferred from neighbouring sites
*wR*(*F*^2^) = 0.117	H-atom parameters not refined
*S* = 1.02	*w* = 1/[σ^2^(*F*_o_^2^) + (0.0561*P*)^2^ + 0.1933*P*] where *P* = (*F*_o_^2^ + 2*F*_c_^2^)/3
4041 reflections	(Δ/σ)_max_ < 0.001
219 parameters	Δρ_max_ = 0.27 e Å^−^^3^
0 restraints	Δρ_min_ = −0.19 e Å^−^^3^

### Special details

**Table:**

Experimental. Absorption corrections were made using the program *SADABS* (Sheldrick, 1996)
Geometry. All e.s.d.'s (except the e.s.d. in the dihedral angle between two l.s. planes) are estimated using the full covariance matrix. The cell e.s.d.'s are taken into account individually in the estimation of e.s.d.'s in distances, angles and torsion angles; correlations between e.s.d.'s in cell parameters are only used when they are defined by crystal symmetry. An approximate (isotropic) treatment of cell e.s.d.'s is used for estimating e.s.d.'s involving l.s. planes.
Refinement. Refinement of *F*^2^ against ALL reflections. The weighted *R*-factor *wR* and goodness of fit *S* are based on *F*^2^, conventional *R*-factors *R* are based on *F*, with *F* set to zero for negative *F*^2^. The threshold expression of *F*^2^ > σ(*F*^2^) is used only for calculating *R*-factors(gt) *etc*. and is not relevant to the choice of reflections for refinement. *R*-factors based on *F*^2^ are statistically about twice as large as those based on *F*, and *R*- factors based on ALL data will be even larger.

### Fractional atomic coordinates and isotropic or equivalent isotropic displacement parameters (Å^2^)

**Table:**

	*x*	*y*	*z*	*U*_iso_*/*U*_eq_	
O1	0.82930 (15)	0.95042 (11)	0.39263 (5)	0.0427 (3)	
C1	0.73618 (16)	0.76549 (14)	0.31531 (6)	0.0251 (3)	
O2	0.47131 (11)	0.66412 (10)	0.32726 (5)	0.0325 (2)	
C2	0.73692 (18)	0.71286 (16)	0.25441 (7)	0.0313 (3)	
H2	0.6815	0.6280	0.2406	0.038*	
O3	0.99412 (11)	0.65133 (9)	0.42002 (5)	0.0279 (2)	
C3	0.8170 (2)	0.78202 (17)	0.21389 (7)	0.0371 (4)	
H3	0.8166	0.7445	0.1727	0.045*	
C4	0.8974 (2)	0.90572 (18)	0.23344 (7)	0.0399 (4)	
H4	0.9525	0.9533	0.2057	0.048*	
C5	0.8977 (2)	0.96002 (17)	0.29298 (8)	0.0407 (4)	
H5	0.9520	1.0454	0.3065	0.049*	
C6	0.81755 (19)	0.88880 (15)	0.33336 (7)	0.0312 (3)	
C7	0.66711 (16)	0.76469 (14)	0.42281 (6)	0.0246 (3)	
C8	0.59500 (16)	0.71138 (16)	0.47021 (7)	0.0296 (3)	
H8	0.5380	0.6254	0.4630	0.036*	
C9	0.60486 (18)	0.78131 (17)	0.52757 (7)	0.0365 (4)	
H9	0.5537	0.7442	0.5591	0.044*	
C10	0.6899 (2)	0.90574 (17)	0.53863 (7)	0.0396 (4)	
H10	0.6972	0.9540	0.5780	0.047*	
C11	0.7638 (2)	0.95994 (16)	0.49306 (7)	0.0380 (4)	
H11	0.8230	1.0450	0.5009	0.046*	
C12	0.75102 (18)	0.88861 (15)	0.43524 (7)	0.0299 (3)	
C13	0.65042 (16)	0.68674 (14)	0.35959 (6)	0.0241 (3)	
C14	0.72913 (16)	0.54401 (13)	0.37383 (6)	0.0233 (3)	
C15	0.90553 (16)	0.53114 (13)	0.40499 (6)	0.0228 (3)	
C16	0.98038 (18)	0.40271 (14)	0.41893 (6)	0.0278 (3)	
H16	1.0993	0.3948	0.4406	0.033*	
C17	0.88084 (19)	0.28611 (15)	0.40104 (7)	0.0318 (3)	
H17	0.9321	0.1983	0.4104	0.038*	
C18	0.70841 (19)	0.29672 (15)	0.36984 (7)	0.0336 (3)	
H18	0.6412	0.2164	0.3574	0.040*	
C19	0.63261 (18)	0.42523 (14)	0.35658 (7)	0.0296 (3)	
H19	0.5132	0.4319	0.3354	0.035*	
C20	1.17721 (17)	0.64420 (16)	0.44035 (7)	0.0330 (3)	
H20C	1.2137	0.5977	0.4826	0.050*	
H20A	1.2251	0.7370	0.4444	0.050*	
H20B	1.2188	0.5930	0.4081	0.050*	
C21	0.3706 (2)	0.78492 (18)	0.31020 (8)	0.0416 (4)	
H21A	0.3727	0.8370	0.3497	0.062*	
H21B	0.2517	0.7598	0.2880	0.062*	
H21C	0.4181	0.8412	0.2811	0.062*	

### Atomic displacement parameters (Å^2^)

**Table:**

	*U*^11^	*U*^22^	*U*^33^	*U*^12^	*U*^13^	*U*^23^
O1	0.0741 (8)	0.0255 (5)	0.0355 (6)	−0.0153 (5)	0.0270 (6)	−0.0072 (5)
C1	0.0279 (7)	0.0237 (7)	0.0234 (7)	0.0048 (5)	0.0063 (5)	0.0034 (5)
O2	0.0226 (5)	0.0347 (6)	0.0355 (6)	0.0027 (4)	−0.0002 (4)	0.0000 (5)
C2	0.0361 (7)	0.0310 (8)	0.0253 (7)	0.0053 (6)	0.0054 (6)	−0.0008 (6)
O3	0.0222 (5)	0.0243 (5)	0.0349 (5)	−0.0020 (4)	0.0038 (4)	−0.0015 (4)
C3	0.0445 (8)	0.0451 (9)	0.0234 (7)	0.0123 (7)	0.0121 (6)	0.0038 (7)
C4	0.0473 (9)	0.0440 (10)	0.0330 (8)	0.0056 (7)	0.0188 (7)	0.0121 (7)
C5	0.0581 (10)	0.0297 (8)	0.0391 (9)	−0.0066 (7)	0.0212 (8)	0.0030 (7)
C6	0.0433 (8)	0.0265 (7)	0.0263 (7)	0.0021 (6)	0.0134 (6)	0.0007 (6)
C7	0.0234 (6)	0.0268 (7)	0.0237 (6)	0.0068 (5)	0.0061 (5)	0.0021 (5)
C8	0.0230 (6)	0.0359 (8)	0.0307 (7)	0.0033 (6)	0.0083 (5)	0.0049 (6)
C9	0.0331 (8)	0.0520 (10)	0.0280 (7)	0.0100 (7)	0.0144 (6)	0.0068 (7)
C10	0.0464 (9)	0.0469 (10)	0.0263 (8)	0.0159 (8)	0.0114 (7)	−0.0041 (7)
C11	0.0532 (9)	0.0293 (8)	0.0331 (8)	0.0039 (7)	0.0141 (7)	−0.0072 (7)
C12	0.0391 (8)	0.0263 (7)	0.0268 (7)	0.0037 (6)	0.0128 (6)	0.0010 (6)
C13	0.0220 (6)	0.0242 (7)	0.0246 (7)	0.0007 (5)	0.0035 (5)	−0.0004 (5)
C14	0.0260 (6)	0.0221 (6)	0.0223 (6)	−0.0006 (5)	0.0074 (5)	0.0003 (5)
C15	0.0272 (6)	0.0215 (7)	0.0210 (6)	−0.0017 (5)	0.0087 (5)	−0.0008 (5)
C16	0.0319 (7)	0.0273 (7)	0.0255 (7)	0.0055 (6)	0.0098 (6)	0.0037 (6)
C17	0.0461 (8)	0.0214 (7)	0.0318 (7)	0.0042 (6)	0.0174 (6)	0.0021 (6)
C18	0.0437 (8)	0.0226 (7)	0.0368 (8)	−0.0081 (6)	0.0147 (7)	−0.0039 (6)
C19	0.0305 (7)	0.0289 (7)	0.0291 (7)	−0.0055 (6)	0.0077 (6)	−0.0022 (6)
C20	0.0238 (7)	0.0382 (9)	0.0365 (8)	−0.0024 (6)	0.0071 (6)	−0.0056 (7)
C21	0.0338 (8)	0.0505 (10)	0.0377 (9)	0.0128 (7)	0.0047 (7)	0.0065 (8)

### Geometric parameters (Å, º)

**Table:**

O1—C12	1.3769 (17)	C9—H9	0.9500
O1—C6	1.3796 (17)	C10—C11	1.375 (2)
C1—C6	1.376 (2)	C10—H10	0.9500
C1—C2	1.3979 (19)	C11—C12	1.396 (2)
C1—C13	1.5202 (18)	C11—H11	0.9500
O2—C21	1.4230 (18)	C13—C14	1.5279 (18)
O2—C13	1.4412 (15)	C14—C19	1.3904 (18)
C2—C3	1.385 (2)	C14—C15	1.4052 (18)
C2—H2	0.9500	C15—C16	1.3890 (18)
O3—C15	1.3668 (15)	C16—C17	1.387 (2)
O3—C20	1.4250 (16)	C16—H16	0.9500
C3—C4	1.382 (2)	C17—C18	1.375 (2)
C3—H3	0.9500	C17—H17	0.9500
C4—C5	1.375 (2)	C18—C19	1.391 (2)
C4—H4	0.9500	C18—H18	0.9500
C5—C6	1.393 (2)	C19—H19	0.9500
C5—H5	0.9500	C20—H20C	0.9800
C7—C12	1.377 (2)	C20—H20A	0.9800
C7—C8	1.3950 (18)	C20—H20B	0.9800
C7—C13	1.5221 (18)	C21—H21A	0.9800
C8—C9	1.384 (2)	C21—H21B	0.9800
C8—H8	0.9500	C21—H21C	0.9800
C9—C10	1.384 (2)		
			
C12—O1—C6	118.82 (11)	C7—C12—C11	121.65 (14)
C6—C1—C2	117.45 (13)	O2—C13—C1	110.21 (10)
C6—C1—C13	122.10 (12)	O2—C13—C7	109.83 (10)
C2—C1—C13	120.44 (12)	C1—C13—C7	110.48 (11)
C21—O2—C13	115.17 (11)	O2—C13—C14	105.37 (10)
C3—C2—C1	121.25 (14)	C1—C13—C14	110.54 (10)
C3—C2—H2	119.4	C7—C13—C14	110.30 (11)
C1—C2—H2	119.4	C19—C14—C15	118.33 (12)
C15—O3—C20	117.61 (11)	C19—C14—C13	122.35 (12)
C4—C3—C2	119.87 (14)	C15—C14—C13	119.32 (11)
C4—C3—H3	120.1	O3—C15—C16	123.72 (12)
C2—C3—H3	120.1	O3—C15—C14	115.69 (11)
C5—C4—C3	119.99 (14)	C16—C15—C14	120.59 (12)
C5—C4—H4	120.0	C17—C16—C15	119.75 (13)
C3—C4—H4	120.0	C17—C16—H16	120.1
C4—C5—C6	119.46 (15)	C15—C16—H16	120.1
C4—C5—H5	120.3	C18—C17—C16	120.47 (13)
C6—C5—H5	120.3	C18—C17—H17	119.8
C1—C6—O1	123.24 (13)	C16—C17—H17	119.8
C1—C6—C5	121.98 (14)	C17—C18—C19	119.91 (13)
O1—C6—C5	114.77 (13)	C17—C18—H18	120.0
C12—C7—C8	117.87 (13)	C19—C18—H18	120.0
C12—C7—C13	122.14 (12)	C14—C19—C18	120.95 (13)
C8—C7—C13	119.99 (12)	C14—C19—H19	119.5
C9—C8—C7	121.30 (14)	C18—C19—H19	119.5
C9—C8—H8	119.3	O3—C20—H20C	109.5
C7—C8—H8	119.3	O3—C20—H20A	109.5
C10—C9—C8	119.48 (14)	H20C—C20—H20A	109.5
C10—C9—H9	120.3	O3—C20—H20B	109.5
C8—C9—H9	120.3	H20C—C20—H20B	109.5
C11—C10—C9	120.44 (14)	H20A—C20—H20B	109.5
C11—C10—H10	119.8	O2—C21—H21A	109.5
C9—C10—H10	119.8	O2—C21—H21B	109.5
C10—C11—C12	119.25 (15)	H21A—C21—H21B	109.5
C10—C11—H11	120.4	O2—C21—H21C	109.5
C12—C11—H11	120.4	H21A—C21—H21C	109.5
O1—C12—C7	123.16 (13)	H21B—C21—H21C	109.5
O1—C12—C11	115.19 (13)		
			
C6—C1—C2—C3	0.2 (2)	C2—C1—C13—O2	−58.56 (16)
C13—C1—C2—C3	−178.88 (12)	C6—C1—C13—C7	0.85 (17)
C1—C2—C3—C4	−0.3 (2)	C2—C1—C13—C7	179.88 (11)
C2—C3—C4—C5	−0.1 (2)	C6—C1—C13—C14	−121.52 (14)
C3—C4—C5—C6	0.5 (2)	C2—C1—C13—C14	57.51 (16)
C2—C1—C6—O1	−178.58 (13)	C12—C7—C13—O2	−122.18 (13)
C13—C1—C6—O1	0.5 (2)	C8—C7—C13—O2	57.33 (15)
C2—C1—C6—C5	0.2 (2)	C12—C7—C13—C1	−0.39 (17)
C13—C1—C6—C5	179.28 (13)	C8—C7—C13—C1	179.12 (11)
C12—O1—C6—C1	−2.3 (2)	C12—C7—C13—C14	122.12 (13)
C12—O1—C6—C5	178.78 (13)	C8—C7—C13—C14	−58.37 (15)
C4—C5—C6—C1	−0.6 (2)	O2—C13—C14—C19	−0.14 (17)
C4—C5—C6—O1	178.33 (14)	C1—C13—C14—C19	−119.20 (14)
C12—C7—C8—C9	0.9 (2)	C7—C13—C14—C19	118.33 (14)
C13—C7—C8—C9	−178.59 (12)	O2—C13—C14—C15	179.34 (11)
C7—C8—C9—C10	−0.9 (2)	C1—C13—C14—C15	60.29 (15)
C8—C9—C10—C11	0.1 (2)	C7—C13—C14—C15	−62.19 (15)
C9—C10—C11—C12	0.5 (2)	C20—O3—C15—C16	11.56 (18)
C6—O1—C12—C7	2.8 (2)	C20—O3—C15—C14	−168.57 (11)
C6—O1—C12—C11	−177.83 (13)	C19—C14—C15—O3	179.21 (12)
C8—C7—C12—O1	179.04 (13)	C13—C14—C15—O3	−0.29 (17)
C13—C7—C12—O1	−1.4 (2)	C19—C14—C15—C16	−0.92 (19)
C8—C7—C12—C11	−0.3 (2)	C13—C14—C15—C16	179.58 (12)
C13—C7—C12—C11	179.25 (13)	O3—C15—C16—C17	−179.11 (12)
C10—C11—C12—O1	−179.81 (13)	C14—C15—C16—C17	1.02 (19)
C10—C11—C12—C7	−0.5 (2)	C15—C16—C17—C18	−0.3 (2)
C21—O2—C13—C1	−61.78 (14)	C16—C17—C18—C19	−0.6 (2)
C21—O2—C13—C7	60.16 (15)	C15—C14—C19—C18	0.1 (2)
C21—O2—C13—C14	178.95 (11)	C13—C14—C19—C18	179.55 (13)
C6—C1—C13—O2	122.41 (14)	C17—C18—C19—C14	0.7 (2)

### Hydrogen-bond geometry (Å, º)

Cg is the centroid of the C14–C19 ring.

**Table:**

*D*—H···*A*	*D*—H	H···*A*	*D*···*A*	*D*—H···*A*
C17—H17···O1^i^	0.95	2.55	3.303 (2)	136
C19—H19···O2	0.95	2.29	2.663 (1)	102
C20—H20*C*···*Cg*^ii^	0.98	2.82	3.6802 (16)	147

Symmetry codes: (i) *x*, *y*−1, *z*; (ii) −*x*+2, −*y*+1, −*z*+1.
